# Supplementary material for: Recovery time and predictors of severe acute malnutrition in children aged 6–59 months via an outpatient therapeutic program in Borena zone: A prospective cohort study
Source: PLoS One. 2025 Jan 3;20(1):e0313186. doi: 10.1371/journal.pone.0313186 (PMC11698384; doi:10.1371/journal.pone.0313186)
Supplement: S1 Table — (DOCX) [file pone.0313186.s001.docx]

**S1Table. This is the S1 Title.** English Version Questionnaire.

**Consent Information Sheet**

Good morning/Good afternoon. My name is ……………………I came from Dilla University Department of Reproductive Health.

I am working for an investigator doing his research for the in partial fulfillment in the requirements for degree of masters in Reproductive Health at Dila University.

This study aims to determine time to recovery and predictors among children aged 6-59 months with severe acute malnutrition who are managed in outpatient therapeutic feeding program in Borena zone. I would like to follow some OTP cases who are admitted on OTP program their treatment outcomes and time of recovery and its predictors. All information found in the card and observation of measurements will be confidential and will be used to make a general report by researcher. No names will be included in the report and there will be no way to identify you as one of the people who gave information. Information extracted from these documents/cards will give recovery time and its associated factor and extracted data will be used only for research purpose. We would be thankful if you spend some time giving us OTP cards of children treated at OTP and how their treatment outcomes is going on.

Principal Investigator: Abuna Mohammed Cell Phone +251916609660/+251912053107

Checklist ID…………………

Data collector’s Name…………………….…Signature……………Date…………………...

Supervisor Name…………. Signature……………………….

**Thank you for cooperation!**

| **Structured format for data collection.**  **1. Socio-demographic factors** | | | | |
| --- | --- | --- | --- | --- |
| **S/N** | **Questions** | **Response categories** | | **Remark** |
| 1 | Sex of patient? | 1. Male 2. Female | |  |
| 2 | Age in months of patient? | _________month | |  |
| 3 | Place of residence | 1.Urban 2.Rural | |  |
| 4 | Marital status | 1. Married 3. Divorced  2. Single 4. Widowed | |  |
| 5 | Mother’s/care givers occupation | 1. House wife 2. Farmer  3. Government employee 4. Merchant 5.Others | |  |
| 6 | Mother’s/care giver education | 1.Illiterate 2.Literate | |  |
| 7 | Family income | _______________ | |  |
| 8 | Distance (time of travel in hour)? | _____________hr | |  |
| 9 | Family size | _____________ | |  |
| 10 | House hold food security status |  | | Attached below |
| **2. Admission Information** | | | | |
|  |  |  |  |  |
| **S/N** | **Questions** | **Response categories** | | **Remark** |
| 2.1 | Admission date | _________________ | |  |
| 2.2 | Bilateral pitting edema | 1. 0 2. +  3. ++ 4. +++ | |  |
| 2.3 | Anthropometric measurement values | MUAC _____________cm  Weight _____________kg  Height _____________cm  WFH/WFL_______z-scor | |  |
| 2.4 | Severe wasting with bilateral oedema | 1. Yes 2. No | |  |
| 2.5 | Type of admission | 1. New admission  2. Re-admission  3. Transfer in ( OTP , SC) | |  |
| **3. Taking Routine Medication** | | | | |
| **S/N** | **Questions** | **Response categories** | **Remark** | |
| 3.1 | Received Amoxicillin at admission? | 1.Yes 2.No |  | |
| 3.2 | Received albendazole/mebendazole? | 1.Yes 2.No | If >= 2 years, on 2^nd^ visit | |
| 3.3 | Received folic acid? | 1.Yes 2.No | If sign of anemia present | |
| 3.4 | Received Measles vaccine on 4^th^ visit? | 1.Yes 2.No | >= 9 months | |
| 3.5 | Received anti-malarial drug? | 1.Yes 2.No | If +ve for malaria | |
| 3.6 | Fully vaccinated? | 1.Yes 2.No |  | |
| 3.7 | Child is Breast feeding? | 1.Yes 2.No |  | |
| **4. Co-morbidity condition** | | |  | |
| **S/N** | **Questions** | **Response categories** | **Remark** | |
| 4.1 | Does the child has any general danger sign-(signs……listed ) | 1.Yes 2.No |  | |
| 4.2 | Does the child have Diarrhea? | 1.Yes 2.No |  | |
| 4.3 | Does the child has Vomiting | 1.Yes 2.No |  | |
| 4.4 | Does the child have Cough? | 1.Yes 2.No |  | |
| 4.5 | Does the child have pneumonia? | 1.Yes 2.No | RR_____bpm,T^0^c_____ | |
| 4.6 | Does the child have blood in stool? | 1.Yes |  | |
| 4.7 | Anemia present at admission? | 1.Yes 2.No |  | |
| 4.8 | Skin infections of child present at admission? | 1.Yes 2.No | Specify skin condition_____________ | |
| **5. Types of Malnutrition** | | | | |
| **S/N** | **Questions** | **Response categories** | | **Remark** |
| 5.1 | Edematous | 1.Yes 2.No | |  |
| 5.2 | Non-edematous | 1. Yes 2. No | |  |
| **6. Discharge information** | | | | |
| 6.1 | Date of discharge | ______________________ | |  |
| 6.2 | Anthropometric measurement values | MUAC _____________cm  Weight _____________kg  Height _____________cm  WFH/WFL _______z-score | | Weight gain____  LoS _________ |
| 6.3 | Bilateral pitting edema | 1. 0 2. +  3. ++ 4. +++ | |  |
| 6.4 | Discharge outcome status | 1.Recovered | | |
|  |  | 2. Defaulted | | |
|  |  | 3. Died | | |
|  |  | 4.Non-responder | | |
|  |  | 5. Transfer out | | |

**PARTII: DIETARY DIVERSITY ASSESSMENT QUESTIONNAIRE**

What are the foods and liquids you had yesterday during the day or at night, either separately or combined with other foods or liquids?

| Question Number | Food group , Examples | YES=1  NO=0 |
| --- | --- | --- |
| DDM1 | Any porridge or gruel (made from grains other than teffany cerifam, fafa, milupa, babylac, mother's choice or other commercially fortified baby food? |  |
| DDM 2 | Bread, pasta, rice, noodles, biscuits, cookies or any other food made from oats, maize, barley, wheat, sorghum, millet, or other grain? |  |
| DDM 3 | Any food made from teff, like injera, kita, or porridge? |  |
| DDM 4 | Any white potatoes, white yams, bulla, kocho, cassava, or any other foods made from  roots? |  |
| DDM 5 | Any pumpkin, carrots, squash, or sweet potatoes that are yellow or orange inside? |  |
| DDM 6 | Any dark green, leafy vegetables like kale, spinach or amaranth leaves? |  |
| DDM 7 | Any liver, kidney, heart or other organ meats? |  |
| DDM 8 | Any beef, pork, lamb, goat, rabbit [or wild game meat such as antelope or deer]? |  |
| DDM 9 | Any chicken, duck or other birds? |  |
| DDM 10 | Any eggs? |  |
| DDM 11 | Any fresh or dried fish or shellfish? |  |
| DDM 12 | Any foods made from beans, peas, lentils or pulses? |  |
| DDM 13 | Any nuts or seeds such as peanuts, sesame or sunflower seeds? |  |
| DDM 14 | Any cheese, yogurt, milk or other milk products |  |
| DDM 15 | Any foods made with oil, fat, or butter? |  |
