# Supplementary material for: Recovery time and predictors of severe acute malnutrition in children aged 6–59 months via an outpatient therapeutic program in Borena zone: A prospective cohort study
Source: PLoS One. 2025 Jan 3;20(1):e0313186. doi: 10.1371/journal.pone.0313186 (PMC11698384; doi:10.1371/journal.pone.0313186)
Supplement: Attachment — Submitted filename: PONE-D-24-07649_reviewer N.pdf [file pone.0313186.s002.pdf]

PLOS ONE

Recovery Time and Predictors of Severe Acute Malnutrition in Children Aged 6–59 Months via an Outpatient Therapeutic Program in Borena Zone: A Prospective Study.

--Manuscript Draft--

**Manuscript Number:**	PONE-D-24-07649
**Article Type:**	Research Article
**Full Title:**	Recovery Time and Predictors of Severe Acute Malnutrition in Children Aged 6–59 Months via an Outpatient Therapeutic Program in Borena Zone: A Prospective Study.
**Short Title:**	A Prospective Study.
**Corresponding Author:**	Girma Tenkolu Bune, Phd<br>Dilla University College of Health Sciences<br>Dilla, Gedeo zone ETHIOPIA
**Keywords:**	Amoxicillin; Recovery Time; Time; Therapeutic; SAM; children; Sever; Malnutrition; Acute; Edema; Outpatient; Program; OTP; Borena; Oromia; Southern Ethiopia
**Abstract:**	Background<br><br>Severe acute malnutrition (SAM) is a severe condition causing bilateral pitting edema or signs of wasting in children, with a high mortality risk. An outpatient therapeutic program is recommended for managing SAM children without complications, but there is limited information on recovery time and its determinants.<br><br>Objective<br><br>This study aims to assess the time to recovery and its predictors among children aged 6-59 months with SAM admitted to the Outpatient therapeutic program in the Borena zone, Oromia region, Southern Ethiopia in 2023.<br><br>Methods<br><br>A prospective follow-up study was carried out from March 1–30, 2023, on 322 children aged 6-59 months in health facilities in the Borena zone. After being collected using a structured questionnaire, the data was imported into Epi Data Manager version 3.1 and exported to SPSS version 26 for analysis. Model fitness was assessed using the log rank test, and time-to-recovery from SAM was found to be predicted by Cox regression analysis. Lastly, the study used the Adjusted Hazard Ratio with a p-value < 0.05 and a 95% confidence interval to describe the connection.<br><br>Results<br><br>The median duration for recovery was found to be 42 days with an interquartile range of 35 to 49. Children who received Amoxicillin had a four-times higher recovery rate (AHR=4.09; 95% CI: 2.75, 6.07). The presence of diarrhea prolonged the recovery time by 53.0% (AHR=0.47; 95% CI: 0.36, 0.62), while vomiting prolonged the recovery time by 58.0% (AHR=0.42; 95% CI: 0.32, 0.55). Edema reduced the chances of recovery by 48% (AHR=0.52; 95% CI: 0.36, 0.62).<br><br>Conclusion<br><br>The study concluded that amoxicillin administration on admission significantly improved recovery time, while diarrhea, vomiting, and edema signs negatively affected it. Accordingly,the government should prioritize administering amoxicillin and training medical staff in managing SAM, including early identification of co-morbid conditions.
**Order of Authors:**	Girma Tenkolu Bune, Phd
	Abuna Mohammed, MpH/RH
	Samrawit Hailu, MpH/RH

Powered by Editorial Manager® and ProduXion Manager® from Aries Systems Corporation

	Eden Ashenafi, MpH/RH
**Additional Information:**	
**Question**	**Response**
**Financial Disclosure**<br><br>Enter a financial disclosure statement that describes the sources of funding for the work included in this submission. Review the submission guidelines for detailed requirements. View published research articles from *PLOS ONE* for specific examples.<br><br>This statement is required for submission and **will appear in the published article** if the submission is accepted. Please make sure it is accurate.<br><br>**Funded studies**<br>Enter a statement with the following details:<br>• Initials of the authors who received each award<br>• Grant numbers awarded to each author<br>• The full name of each funder<br>• URL of each funder website<br>• Did the sponsors or funders play any role in the study design, data collection and analysis, decision to publish, or preparation of the manuscript?<br><br>Did you receive funding for this work?	The author(s) received no specific funding for this work.
**Competing Interests**<br><br>Use the instructions below to enter a competing interest statement for this submission. On behalf of all authors, disclose any competing interests that could be perceived to bias this work—acknowledging all financial support and any other relevant financial or non-financial competing interests.<br><br>This statement is required for submission and **will appear in the published article** if the submission is accepted. Please make sure it is accurate and that any funding	The authors have declared that no competing interests exist.

sources listed in your Funding Information later in the submission form are also declared in your Financial Disclosure statement.<br><br>View published research articles from *PLOS ONE* for specific examples.<br><br>**NO authors have competing interests**<br><br>Enter: *The authors have declared that no competing interests exist.*<br><br>**Authors with competing interests**<br><br>Enter competing interest details beginning with this statement:<br><br>*I have read the journal's policy and the authors of this manuscript have the following competing interests: [insert competing interests here]*<br><br>* typeset	
**Ethics Statement**<br><br>Enter an ethics statement for this submission. This statement is required if the study involved:<br><br>• Human participants<br>• Human specimens or tissue<br>• Vertebrate animals or cephalopods<br>• Vertebrate embryos or tissues<br>• Field research<br><br>Write "N/A" if the submission does not require an ethics statement.<br><br>General guidance is provided below. Consult the submission guidelines for detailed instructions. **Make sure that all information entered here is included in the Methods section of the manuscript.**	The study process included several measures to ensure ethical standards were upheld. Prior to initiating the study, ethical approval was granted by the Institutional Review Board (IRB) at Dila University's College of Health Sciences and Medicine, followed by the IRB coordinator sending an official letter to the Borena zone health office. Subsequently, the Borena zone health office forwarded similar communication to the district health offices and OTP site, obtaining verbal consent from the mothers or caregivers while emphasizing the importance of data confidentiality and security. Noteworthy is the fact that the data extraction form did not include the names of the children enrolled in the OTP service to safeguard their privacy. Verbal consent was also obtained from the mothers or caregivers, and stringent measures were put in place to ensure the confidentiality and security of the data. The study strictly followed ethical guidelines and regulations to ensure the safety and well-being of all participants involved in the research.

**Format for specific study types**<br><br>**Human Subject Research (involving human participants and/or tissue)**<br>• Give the name of the institutional review board or ethics committee that approved the study<br>• Include the approval number and/or a statement indicating approval of this research<br>• Indicate the form of consent obtained (written/oral) or the reason that consent was not obtained (e.g. the data were analyzed anonymously)<br><br>**Animal Research (involving vertebrate animals, embryos or tissues)**<br>• Provide the name of the Institutional Animal Care and Use Committee (IACUC) or other relevant ethics board that reviewed the study protocol, and indicate whether they approved this research or granted a formal waiver of ethical approval<br>• Include an approval number if one was obtained<br>• If the study involved *non-human primates*, add *additional details* about animal welfare and steps taken to ameliorate suffering<br>• If anesthesia, euthanasia, or any kind of animal sacrifice is part of the study, include briefly which substances and/or methods were applied<br><br>**Field Research**<br><br>Include the following details if this study involves the collection of plant, animal, or other materials from a natural setting:<br>• Field permit number<br>• Name of the institution or relevant body that granted permission	
**Data Availability**<br><br>Authors are required to make all data underlying the findings described fully available, without restriction, and from the time of publication. PLOS allows rare exceptions to address legal and ethical concerns. See the PLOS Data Policy and FAQ for detailed information.	Yes - all data are fully available without restriction

Powered by Editorial Manager® and ProduXion Manager® from Aries Systems Corporation

A Data Availability Statement describing where the data can be found is required at submission. Your answers to this question constitute the Data Availability Statement and **will be published in the article**, if accepted.<br><br>**Important:** Stating 'data available on request from the author' is not sufficient. If your data are only available upon request, select 'No' for the first question and explain your exceptional situation in the text box.<br><br>Do the authors confirm that all data underlying the findings described in their manuscript are fully available without restriction?	
**Describe where the data may be found in full sentences. If you are copying our sample text, replace any instances of XXX with the appropriate details.**<br><br>• If the data are **held or will be held in a public repository**, include URLs, accession numbers or DOIs. If this information will only be available after acceptance, indicate this by ticking the box below. For example: *All XXX files are available from the XXX database (accession number(s) XXX, XXX.).*<br>• If the data are all contained **within the manuscript and/or Supporting Information files**, enter the following: *All relevant data are within the manuscript and its Supporting Information files.*<br>• If neither of these applies but you are able to provide **details of access elsewhere**, with or without limitations, please do so. For example:<br><br>*Data cannot be shared publicly because of [XXX]. Data are available from the XXX Institutional Data Access / Ethics Committee (contact via XXX) for researchers who meet the criteria for access to confidential data.*<br><br>*The data underlying the results presented in the study are available from (include the name of the third party*	All relevant data is within the manuscript and its supporting information files.

and contact information or URL).<br>• This text is appropriate if the data are owned by a third party and authors do not have permission to share the data.<br><br>* typeset	
Additional data availability information:	

Manuscript
Click here to access/download;Manuscript;Body manuscript for PLOSEONE.docx

Recovery Time and Predictors of Severe Acute Malnutrition in Children Aged 6–59 Months via an Outpatient Therapeutic Program in Borena Zone: *A Prospective Study.*

Abuna Mohammed[1], Girma Tenkolu Bune(PhD)*[2], Samrawit Hailu,[3]Eden Ashenafi[4]

Authors' Affiliations and Address

[1]Department of Reproductive Health, College of Health and Medical Science, Dilla University, Dilla, Ethiopia. Email: sarabuna2010@gmail.com

[2]School of Public Heath, College of Health and Medical Sciences, Dilla University, Dilla, Ethiopia. Email: girmatbune@gmail.com

[2]School of Public Heath, College of Health and Medical Sciences, Dilla University, Dilla, Ethiopia.

[4]Department of Reproductive Health, College of Health and Medical Science, Dilla University, Dilla, Ethiopia. Email: eden.ashenafi@du.edu.et

[*1]Corresponding Author:

Dr. Girma Tenkolu Bune, Email Address: girmatbune@gmail.com

Abstract:

Background: Severe acute malnutrition (SAM) is a severe condition causing bilateral pitting edema or signs of wasting in children, with a high mortality risk. An outpatient therapeutic program is recommended for managing SAM children without complications, but there is limited information on recovery time and its determinants.

Objective: This study aims to assess the time to recovery and its predictors among children aged 6-59 months with SAM admitted to the Outpatient therapeutic program in the Borena zone, Oromia region, Southern Ethiopia in 2023.

Methods: A prospective follow-up study was carried out from March 1–30, 2023, on 322 children aged 6-59 months in health facilities in the Borena zone. After being collected using a structured questionnaire, the data was imported into Epi Data Manager version 3.1 and exported to SPSS version 26 for analysis. Model fitness was assessed using the log rank test, and time-to-recovery from SAM was found to be predicted by Cox regression analysis. Lastly, the study used the Adjusted Hazard Ratio with a p-value < 0.05 and a 95% confidence interval to describe the connection.

Results: The median duration for recovery was found to be 42 days with an interquartile range of 35 to 49. Children who received Amoxicillin had a four-times higher recovery rate (AHR=4.09; 95% CI: 2.75, 6.07). The presence of diarrhea prolonged the recovery time by 53.0% (AHR=0.47; 95% CI: 0.36, 0.62), while vomiting prolonged the recovery time by 58.0% (AHR=0.42; 95% CI: 0.32, 0.55). Edema reduced the chances of recovery by 48% (AHR=0.52; 95% CI: 0.36, 0.62).

Conclusion: The study concluded that amoxicillin administration on admission significantly improved recovery time, while diarrhea, vomiting, and edema signs negatively affected it. Accordingly,the government should prioritize administering amoxicillin and training medical staff in managing SAM, including early identification of co-morbid conditions.

Keywords: Amoxicillin, Recovery Time, Time, Therapeutic, SAM, Children, Sever, Malnutrition, Acute, Edema, Outpatient, Program, OTP, Borena, Oromia, Southern Ethiopia

1. Introduction

Recent data from UNICEF, World Bank, and WHO highlights that approximately 13.6 million children under the age of five are affected by severe acute malnutrition (SAM) globally, making up 2.0% of all childhood malnutrition cases in 2020, with a significant portion concentrated in Asia(1). Sub-Saharan Africa is also grappling with a notable challenge of severe wasting, with around 2.5 million cases impacting nearly 3 million children, and projections indicate that by 2030, the region will surpass South Asia in having the highest number of severe acute malnutrition cases worldwide(2).

Ethiopia stands out as a country with the highest prevalence of severe acute malnutrition in Sub-Saharan Africa, with just 1% of children facing severe malnutrition, as per the 2019 EMDHS(3).. However, malnutrition rates vary across different regions within the country, with areas like Somalia, Afar, and Gambela experiencing higher rates compared to Addis Ababa and Harari. Despite a decrease in wasting prevalence

over the past two decades, regions affected by desert locusts have witnessed a concerning 20% rise in severe acute malnutrition cases between January and February 2020(1,4).

Severe acute malnutrition remains a significant contributor to child mortality globally, causing .approximately one million deaths annually, particularly in low and middle-income nations. In Ethiopia, malnutrition-related factors account for 57% of child deaths(5). The government has been implementing various strategies to combat this issue, including improving food availability through economic growth and agricultural initiatives, as well as enhancing early warning systems and emergency response services. However, challenges such as shorter recovery times for severe acute malnutrition cases persist due to factors like case management and household dynamics (6).

In the Ethiopian Borena region, 7000 cases of severe acute malnutrition were recorded between July and June 2022; 97% of the affected children recovered. The fact that 85.7% of these individuals were admitted to outpatient therapeutic feeding programs suggests that malnutrition is being addressed proactively(7). However, certain children needed longer care than others, which could put families in a difficult financial situation. The average expense per children receiving treatment for malnutrition was US$134.88, underscoring the financial burden associated with long-term (8). Prior research in this field has been limited in scope and has not provided a comprehensive examination of the various elements influencing malnutrition rates. Insufficient research has been done

on factors such as household food security, family size, income levels, and maternal education, which has led to malnutrition in Ethiopia's Borena zone.

Acknowledging the critical nature of the circumstances and the necessity for additional examination, this upcoming longitudinal study seeks to evaluate the duration of recovery and the factors influencing it among children suffering from severe acute malnutrition in the Borena Zone, Southern Ethiopia. This study is crucial in providing insights into the influence of various factors on recovery outcomes, which are essential for shaping future healthcare policies and public health initiatives in Ethiopia, particularly in regions prone to severe droughts. Additionally, it will enhance the existing knowledge base and provide researchers and the global scientific community with additional resources to advance research in this area.

2. Materials and Methods

Study Area and Period

The study was conducted in the Borena zone of the Oromia Region in Ethiopia. The zone consists of 13 woredas, with its capital city being Yabelo Town, located 567 km from Addis Ababa in the Southern part of the country. Borena is bordered by Kenya to the south, the SNNPR to the west, the West Guji and Guji zones to the north, and the Dawa zone of the Somali Region to the east. The zone has a total of five hospitals, 45 health centers, 165 health posts, and 70 private clinics. The population of the zone is 793,340, with 16.43% (130,346) of them being under five years old, and 15% (119,001) being children aged 6 to 59 months. From July 2021 to June 2022, a total of 6,005 children with severe acute malnutrition (SAM) were enrolled in an outpatient therapeutic feeding program in this zone. (7) Throughout the zone, there are 175 functional OTP

sites that offer community-based treatment for severe acute malnutrition. The study participants were enrolled from March 1-30, 2023, and followed weekly until June 30, 2023. (Fig.1.See sketch Map of Borena Zone)

Study design

A prospective follow-up design was employed in this institution-based study.

Study population and Inclusion and Exclusion Criteria

The study population included all children aged 6 to 59 months in the Borena zone who had uncomplicated severe acute malnutrition, passed an appetite test, and were enrolled in the Outpatient Treatment Program (OTP). Specifically, children who met the inclusion criteria and were enrolled in a randomly chosen OTP service site during the study period were considered as study participants. All OTP admissions enrolled from March 1-30, 2023, were included in the study. Children aged 6-59 months who met the admission requirements of the OTP program (i.e., bilateral pitting edema, WFL/WFH less than -3 Z-score, and no medical complications with a passed appetite test) were included in the study. Children admitted to the OTP program based on clinical judgment, those with congenital defects that prevent accurate measurement of anthropometric indices, those transferred from stabilization centers, and those with known chronic conditions were excluded from the study.

Sample size Determination

To determine the sample size, we utilized the Epi Info version TM 7.2.5.0 software, employing a multi-stage sampling method and assuming a 95% confidence interval,

80% power, and a one-to-one exposed to unexposed ratio(9). For the first objective, we used the program performance indicator of the frequency of children who recovered among those admitted (65.3%), as well as an adjusted hazard ratio (AHR) of 0.81 for the second objective, representing the effect of having diarrhea on the cure rate compared to not having diarrhea. Additionally, we considered the proportion of the outcome in the unexposed group, which was 88.18% among severely malnourished children enrolled in the OTP program in North Gonder, Ethiopia. (9) Using the Epi Info software, we initially calculated a sample size of 204 for the second objective; however, we included a 5% compensation to account for potential loss to follow-up and adjusted for a design effect of 1.5 due to the multi-stage sampling method used. As a result, the final sample size for this study was determined to be 322. With this sample size, we anticipate having sufficient statistical power to identify significant associations between the predictors and the duration of recovery for children enrolled in the OTP program. (Table 1)

Table 1: Significant determinant of time to recovery from SAM among children 6-59 months used to calculate the largest sample size for the second objective (17, 18)

**Variable**	**% of outcome in unexposed group**	**AHR**	**Sample size by the second objective**	**Drop outs**	**Design effect**	**Final sample size**	**Reference**
**Mother travel < 30 minutes to**	19.9	2.6	82	5%	1.5	129	Amare B et al, 2021.

**service site**							
**Children treated by trained HEW**	24	2.1	118	5%	1.5	186	
**Taking Amoxicillin**	32.1	2.3	52	5%	1.5	82	Worku N et al, 2017.
**Children with diarrhea**	88	0.81	204	5%	1.5	322	

Sampling Techniques and procedures

In the Borena zone, which comprises 13 districts and 175 functional OTP service sites, the sampling process was conducted in two stages. In the first stage, a lottery method was used to randomly select five districts from the total. Within these selected districts, there were 108 OTP sites. Moving to the second stage, 34 OTP sites were then randomly chosen from each of the selected districts using the same lottery method. This selection was carried out to ensure representativeness, targeting approximately 30% of the OTP sites within each district. Population proportion-to-size allocation was utilized to guarantee proportional representation. Due to the small scale of the study, all eligible children with severe acute malnutrition (SAM) were consecutively included from the randomly selected OTP sites. These children were followed up until the occurrence of the outcome, allowing for a comprehensive understanding of the duration of recovery for children in the OTP program and the factors influencing this period. By employing this sampling strategy, the study aims to encompass a diverse range of OTP sites and offer valuable insights into the population of children with severe acute malnutrition in the Borena zone. (Fig.2.Schematic diagram of sampling technique and procedure)

Data Collection, Methods and Instruments

The interviewer used a pre-tested, structured questionnaire and had a language expert translate it into Afan Oromo and back into English to ensure consistency. Data was collected by six trained diploma nurses with prior experience in data collection, who conducted in-person interviews with mothers or caregivers to gather information on socio-demographic characteristics and household food variety. Anthropometric measures (weight and height/length) and physical examinations were used to identify signs of malnutrition in study participants. Weight was recorded weekly for children under two years using a calibrated 25 kg hanging scale, with measurements reported to the nearest 0.1 kg. A standardized SECA scale was used for children over two years, with calibration and accuracy checks carried out regularly. Measurements of length and height were taken using a wooden measuring table and stadiometer, with specific procedures for different age groups. Medical complications and signs of malnutrition were assessed during enrollment and subsequent follow-ups using a standard OTP treatment card. The desire to eat assessment was conducted in a calm, private setting, and a child was considered to have passed the appetite test if they consumed part of the RUTF over thirty minutes. (11). Severely acute malnourished (SAM) children meeting specific criteria were included in the study and followed up weekly, with health and anthropometric data captured at each visit. Participants visited the site weekly for management and examination by the service provider and data collectors.

Study Variables

Dependent variable: Time to recovery: measured in days from the date the SAM child admitted on OTP to the date the child was discharged as recovered (i.e. Absence of bilateral pitting edema for two uninterrupted visits AND WFH/WFL greater or equal to -2 z-score and clinically in good condition and attentive) is considered as dependent variables. Children who were discharged from treatment but had not fully recovered, such as those who were transferred out, defaulted, died, or did not respond, were considered as censored.

Independent variables: Socio- demographic factors include age, sex, residence place, mother's education level, mother's occupation, marital status, distance from OTP site, house hold food security status, and family size; **Co-morbid conditions (presence):** Diarrhea, vomiting, cough, skin infection, blood in stool, anemia (palmar pallor); **Routine medication:** Providing Amoxicillin, de-worming tablet**,** folic acid; **Types of severe acute malnutrition:** Edematous, Non-edematous (Marasmus); **Service provision related variables:** Key message provision, SAM child referral way to OTP

Operational definition

OTP admission criteria: Bilateral pitting edema (grade + or ++) OR WFL/WFH less than -3 Z-score AND absence of medical complications, passed desire to eat and clinically good and vigilant. (6) **Routine medication:** Children older than two years will receive deworming tablet during their second visit. If anemia symptoms are present, a 5 mg dose of folic acid will be given once and amoxicillin will be administered upon admission for all SAM children for seven days (6) **Recovered**: is cured fully in OTP- (If admitted by edema: Absence of bilateral pitting edema for 2 successive visits and WFH/WFL greater or equal to -2 z-score and clinically good and vigilant. If enrolled based on

WFH/WFL, discharged cured when: WFH/WFL greater or equal to -2 z-score , absence of bilateral pitting edema and clinically good and vigilant)(6). **Event**: Refers to the recovery of severely malnourished children while discharged from the program. **Censored:** in this study included malnourished children who did not recover due to transfer out, default, death, or non-responding. **Time to recovery**: was measured in days from the child admitted by SAM on OTP to the child discharged as recovered. **Chronic diseases**: TB, HIV, DM, Chronic heart diseases(11). **Household Food Diversity Score:** was assessed by using standard tools (7 number of food groups questions assessed) and computed by SPSS software, those who scored ≥ 4.5 were considered as medium dietary diversity, and those below < 4.5 were regarded as poor dietary diversity (12). **Key Messages of OTP:** - Deliver the following instructions to the caregivers: inform them about the appropriate timing and method of administering medication to the child, guide them on how to give Ready-to-Use Therapeutic Food (RUTF) for the child, educate them on day to return to outpatient care, and emphasize the importance of taking the child to the hospital immediately if their condition worsens.(6)

Data Processing and Analysis

The data collected was coded, edited, and entered into Epi data version 3.1 before being exported and analyzed using SPSS version 26. Descriptive analysis was carried out to assess missing data, outliers, normality, and multi-collinearity. The Kaplan-Meier survival curve was used to compare recovery times among different independent categorical variables, and the model's fitness was evaluated using the log-rank (Mantel-Cox) test and graphical assessment of proportional hazards over time. Cox regression analysis was utilized to identify factors affecting recovery time from severe acute

malnutrition, with adjusted Hazard Ratio and 95% confidence interval determining significant associations. The study's findings were presented through tables, figures, charts, and descriptions.

Data Quality assurance

To ensure data quality, the collection tool was adapted to address study variables, and a pre-test was conducted on 5% of the sample in another district before actual data collection. Training was provided to data collectors and supervisors, and data collection was closely monitored by the principal investigator and supervisors. Thorough review and daily checks were conducted to ensure data completeness and accuracy.

Ethical Consideration

The study process included several measures to ensure ethical standards were upheld. Prior to initiating the study, ethical approval was granted by the Institutional Review Board (IRB) at Dila University's College of Health Sciences and Medicine, followed by the IRB coordinator sending an official letter to the Borena zone health office. Subsequently, the Borena zone health office forwarded similar communication to the District health offices and OTP site, obtaining verbal consent from the mothers or caregivers while emphasizing the importance of data confidentiality and security. Noteworthy is the fact that the data extraction form did not include the names of the children enrolled in the OTP service to safeguard their privacy. Verbal consent was also obtained from the mothers or caregivers, and stringent measures were put in place to ensure the confidentiality and security of the data. The study strictly followed ethical guidelines and regulations to ensure the safety and well-being of all participants involved in the research.

3. Results

Socio-demographic characteristics of participants

The study involved 322 individuals who all participated, resulting in a 100% response rate. The average age of the study subjects was 22.6 months, with a standard deviation of ± 14.6. Out of the 322 participants, 211 (65.5%) were aged <= 24 months, and slightly over half, 167 (51.9%), were male. The majority of the participants, 265 (82.3%), resided in rural areas. In terms of family size, most children, 290 (90.1%), belonged to families with less than 5 members. Additionally, more than 82.3% of the participants traveled less than 30 minutes to reach OTP sites (Table2).

Table 2: Socio-demographic characteristics of caretakers and SAM managed at OTP in Borena zone, Southern Ethiopia 2023 (n=322)

Variables	Frequency	%
**Sex**		
Male	167	51.9
Female	155	48.1
**Age in month**		
≤ 24	211	65.5
> 24	111	34.5
**Place of residence**		
Rural	265	82.3
Urban	57	17.7
Family size		
< 5	290	90.1

≥ 5	32	9.9
**Caretakers educational status**		
Unable to read and write	155	48.1
Primary	51	15.8
Secondary and above	116	36
**Marital status of the mother/caregiver**		
Married	294	91.3
Single	9	2.8
Divorced	9	2.8
Widowed	10	3.1
**Mother's/caregivers' occupation**		
Housewife	220	68.3
Farmer	32	9.9
Government employee	46	14.3
Merchant	24	7.5
**Travelling distance to the health post**		
<30 minutes	269	83.5
>=30 minutes	53	16.5

Household Food Diversity status of Caretakers/mothers and children

The analysis of household food diversity revealed that a significant number of households included grains/cereals and white tubers and roots in their diet.

Nevertheless, there was a noticeable lack of consumption when it came to pulses, nuts, seeds, milk and milk products, as well as other vegetables, as indicated in (Table 3).

Table 3*:* Household food diversity score of caretakers and children aged 6-59 months diagnosed with SAM managed at OTP ram in Borena zone, Southern Ethiopia 2023 (n= 322)

**Variables**	**Frequency**	**%**
Food made from grains/cereals and White tubers and roots		
Yes	284	88.2
No	38	11.8
Pulses, Nuts and seeds (tree nuts and ground		
Yes	59	18.3
No	263	81.7
Milk and milk products		
Yes	187	58.1
No	135	41.9
Organ meat (iron-rich), Flesh meats, Eggs and food made from it.		
Yes	148	46
No	174	54
Dark green leafy vegetables		
Yes	71	22
No	251	78
Vitamin-A rich vegetables and fruits		
Yes	91	28.3

No	231	71.7
Other vegetables		
Yes	102	31.7
No	220	68.3
The overall HHFD score		
<4.5(Poor)	243	75.5
≥ 4.5(Medium and above)	79	24.5

Routine medications provision

In this context, 279 children participating in the research were administered amoxicillin upon admission, representing 86.6% of the total. Conversely, only 28 children, amounting to 14.3%, were given deworming medication during the subsequent monitoring period. These results can be found in (Table 4).

Co-morbidity conditions

Moving on to the co-morbidity conditions, it was observed that 63.7% of the children had acute diarrhea during their admission, while 49.4% experienced vomiting. Furthermore, 31.4% of the children displayed signs of edema upon admission. These findings are presented in (Table 4).

Table 4: Co-morbidities and routine medications provided for children aged 6-59 months diagnosed with SAM managed at OTP ram in Borena zone, Southern Ethiopia 2023 (n= 322)

Variables	Frequency	%
Amoxicillin given during admission		

Yes	279	86.6
No	43	13.4
Deworming		
Yes	28	14.3
No	88	85.7
Diarrhea during admission		
Yes	205	63.7
No	117	36.3
Vomiting during admission		
Yes	159	49.4
No	163	50.6
Cough during admission		
Yes	129	40.1
No	193	59.9
Blood in stool		
Yes	2	0.6
No	320	99.4
Palmar pallor		
Yes	16	5.0
No	306	95.0
Skin infections		
Yes	235	73.0
No	87	27.0

Types of Severe Acute Malnutrition During admission (n=322)

Severe acute malnutrition cases were classified into two types during admission, with 221 cases (68.6%) diagnosed with marasmus and the remaining 101 cases (31.4%) diagnosed with edema, as illustrated in (Fig.3.Types of SAM diagnosed during admission).

Survival status of children

The median follow-up period for children undergoing OTP treatment for severe acute malnutrition was 49 days, ranging from 21 to 112 days. The median recovery duration was 42 days, with an interquartile range of 35 to 49 days. The survival probability during OTP treatment was high, starting at approximately 0.99 on the third day, decreasing to 0.01 by the end of the 112-day follow-up period, as shown in (Fig.4 Survival probability on OTP treatment).

Analysis through the log-rank test demonstrated that children with skin infections had a significantly longer recovery time during OTP treatment compared to those without skin infections ($p < 0.01$), as depicted in (Fig.5.Survival status among SAM children).

Furthermore, the log-rank test revealed that children who received Amoxicillin upon admission for OTP treatment had a significantly shorter recovery time than those who did not receive it ($p < 0.01$), as shown in (Fig. 6. Survival status among SAM children received Amoxicillin).

Treatment outcomes of children with severe acute malnutrition

In the study on treatment outcomes of children with severe acute malnutrition, it was found that 93.2% of the total study subjects successfully recovered from the condition. There were 1.9% cases of default, 2.5% cases of non-response, 0.3% cases of mortality, and 2.2% cases of transfer out.

Predictors of time to recovery

The bivariate analysis identified several factors that can predict the time to recovery. These factors include travel distance from the OTP site, delivery of OTP key messages, household food diversity score, administration of Amoxicillin during admission, deworming, presence of diarrhea, vomiting, skin infection, and edema at admission. A statistical Log rank (Mantel-Cox) test was conducted to check the model appropriateness, which showed a significant difference in survival between the groups with a significance level of $P<0.001$. After adjusting for possible confounders in the multivariable analysis, it was found that children who received Amoxicillin during admission had a 4.09 times higher rate of recovery (AHR=4.09; 95% CI: 2.75, 6.07) compared to those who did not receive it. Children with diarrhea at admission had a 53% longer recovery time (hazard ratio = 0.47; 95% CI = 0.36, 0.62) compared to those without diarrhea. On the other hand, children with vomiting at admission had a 58.0% longer recovery rate (AHR=0.42; 95% CI: 0.32, 0.55) compared to their counterparts. The presence of edema at admission reduced the cure rate by 48% (AHR=0.52; 95% CI: 0.36, 0.62) (Table 5).

Table 5: Predictors of time to recovery among children aged 6-59 months diagnosed with SAM managed at OTP in Borena zone, Oromia, Southern Ethiopia 2023(n=322)

Outcome

Variables	Event	Censored	CHR(95%:CI)	AHR(95%:CI)	P-value
**Traveling distance to Health post**					
**< 30 minute**	251	18	1.45(1.07, 1.97)	0.96(0.69, 1.34)	0.83
**>/= 30 minute**	49	4	1	1	
**Key messages delivered**					
**Yes**	219	16	0.65(0.50,0.84)	1.61(0.22,11.82)	0.64
**No**	81	6	1	1	
**HHFDS**					
**<4.5**	226	17	0.76(0.58,0.98)	0.97(0.74 , 1.28)	0.85
**>/=4.5**	74	5	1	1	
**Amoxicillin at admission**					
**Yes**	262	17	2.48(1.75,3.53)	4.09(2.75, 6.07)	< 0.001
**No**	38	5	1	1	
**Deworming during follow up**					
**Yes**	20	8	1.23(0.87 , 1.70)	0.87(0.61,1.23)	0.42

**No**	76	12	1	1	
**Diarrhea at admission**					
**Yes**	190	15	0.48(0.38,0.62)	0.47(0.36,0.62)	<0.001
**No**	110	7	1	1	
**Vomiting at admission**					
**Yes**	149	10	0.75(0.59,0.94)	0.42(0.32,0.55)	<0.001
**No**	151	12	1		
**Skin infections at admission**					
**Yes**	219	16	0.64(0.49,0.83)	0.85(0.64, 1.13)	0.27
**No**	81	6	1		
**Edema present at admission**					
**Yes**	55	7	0.57(0.42,0.77)	0.52(0.36,0.62)	<0.001
**No**	245	15	1		

4. Discussion

The primary objective of this study was to report on the median recovery time from severe acute malnutrition and analyze the influencing factors in the Borena zone of Oromia region, Southern Ethiopia, in 2023. Among the 322 children monitored, an impressive 93.2% achieved successful recovery from severe acute malnutrition, underscoring the effectiveness of the treatment protocols in place. The longest recovery period observed was 112 days, with no child surpassing this duration, indicating the overall success of the intervention strategies. Meeting and exceeding the international standard of a maximum 60-day average length of stay in therapeutic feeding units study (11), this study recorded a median recovery time of 42 days, falling within the acceptable range set by established norms, with an interquartile range from 35 to 49 days. Factors such as receiving Amoxicillin upon admission and presenting symptoms like diarrhea, vomiting, and edema at admission were identified as significant predictors affecting the recovery duration from severe acute malnutrition.

As per the study report, the median recovery period of 42 days is in line with the worldwide reference standards (Sphere) (11) and the findings of similar studies conducted in Afar, Ethiopia (13), wherein the mean duration of therapy was reported to be 44.15 days, Northeastern Ethiopia and Gondar Province(9,14) In contrast to studies conducted in Bahir Dar City, Northwest Ethiopia(15), our study indicated that participants in the outpatient therapy program stayed longer. These discrepancies in recovery times could be brought about by disparities in the research area's

sociodemographic makeup, treatment and caring practices across facilities, and supply availability.

Our study discovered that the administration of Amoxicillin during hospitalization significantly improved the rate of recovery, which is consistent with research conducted in rural Malawi(16),. and the Afar and North Gondar regions of Ethiopia. (9,13). In these studies, it was found that antibiotics, when used as part of the treatment for severe acute malnutrition, led to increased weight gain and higher recovery rates among children. This association can be attributed to several factors. Firstly, children with severe acute malnutrition often experience bacterial overgrowth in the small bowel, which can result in systemic infection and hinder the absorption of essential nutrients. By incorporating Amoxicillin into the treatment of severe acute malnutrition within outpatient therapeutic feeding programs, the growth of bacteria in the small bowel can be effectively controlled. Therefore, it is crucial to administer antibiotics to all severely malnourished children, even if they do not exhibit clinical symptoms of systemic infection. In reality, most children with severe acute malnutrition are infected, especially if they have a poor appetite and require immediate treatment. Mishandling these infections can further complicate the recovery process for children with severe acute malnutrition. (11)

The results of this study also revealed that patients who had diarrhea upon admission had a recovery time that was 53% longer compared to those who did not have diarrhea. Similarly, children who experienced vomiting at admission had a delay in their recovery rate by 58% when compared to those without vomiting. These findings are consistent

with previous research conducted in North Gondar, Ethiopia, which also found lower recovery rates among children with diarrhea and vomiting upon admission.(9) Another study conducted in Southern Ethiopia further supported these results by indicating that diarrhea as a co-morbidity is associated with a slower recovery from severe acute malnutrition(17) Additionally, a study conducted in Arbaminch Zuria district demonstrated that diarrhea in children was an unfavorable predictor for the duration of recovery from severe acute malnutrition.(18) The observed connection between recovery rates and diarrhea and vomiting may be attributed to the fact that these conditions hinder the absorption of nutrients, leading to an increased demand for nutrients and resulting in unfavorable nutritional outcomes and prolonged recovery times. (11)

Furthermore, the study findings demonstrated a significant correlation between the recovery time from severe acute malnutrition and the different types of severe acute malnutrition present at admission. For instance, this study highlighted that the presence of edema upon admission was associated with a 48% slower recovery rate (AHR = 0.52; 95%CI: 0.36, 0.62). A separate study in Dire Dawa also indicated that children with edema required a longer duration for recovery, while those weighing at least 7 kg upon admission were more likely to recover faster.(19) Additionally, data from the Arbaminch Zuria district suggested that children admitted with edema had an extended stay in the outpatient therapeutic feeding program. The commonality across these studies may be attributed to the likelihood that children meeting the edema criteria upon admission could have more severe nutritional deficiencies and imbalances compared to non-edematous children with severe acute malnutrition.(18) The process of nutritional

rehabilitation, which involves gradually increasing nutrient intake and addressing specific nutrient deficiencies, plays a critical role in the recovery process. It is plausible that this comprehensive nutritional rehabilitation process may require a longer duration for edematous children, ultimately leading to an extended recovery period.

One of the advantages of this study is the utilization of primary data, which can demonstrate cause-and-effect relationships. However, it can be challenging to maintain participant follow-up, resulting in missing data and a decrease in statistical power. The presence of social desirability bias may lead to an overestimation or underestimation of family dietary variety variables based on 24-hour food consumption. To address these limitations, a thorough examination of the data was conducted, and measures were implemented to mitigate potential biases. Regular check-ins with the children on a weekly basis were carried out to minimize delays in determining the desired outcomes, and information regarding the sociodemographic characteristics of mothers and caregivers at the OTP sites was collected throughout the trial.

5. Conclusion

The study result indicated that the time it takes for patients to recover is similar to previous studies. Several factors were identified as predictors of recovery time, including the administration of Amoxicillin upon admission, as well as the presence of symptoms such as diarrhea, vomiting, and edema at the time of admission. Interestingly, the presence of these symptoms upon admission was found to prolong the recovery process, while the timely provision of routine medication like Amoxicillin resulted in faster recovery from severe acute malnutrition. These findings highlight the

importance of healthcare facilities and providers prioritizing the administration of Amoxicillin upon admission for children with severe acute malnutrition in order to expedite their recovery. It is crucial to educate caregivers on medication and nutrition, implement regular monitoring and follow-up protocols, and provide comprehensive training to healthcare professionals on managing severe acute malnutrition, including the early identification of co-morbid conditions like diarrhea, vomiting, and edema. Adequate allocation of resources for therapeutic feeding unit supplies and supportive supervision visits is also necessary. Moreover, further research is required to explore the impact of diarrhea, vomiting, and edema on recovery time.

Acknowledgement:

We would like to thank Dilla University College of Health and Medical Science, School of Public health, Reproductive Health Department for giving us this opportunity and for the overall support provided to undertake this thesis. We would also like to thank Borena Zone Health Office for supplying important data. Finally, but just as particularly, we thank the data collectors, supervisors and study participants for their remarkable input for this study.

References

1. UNICEF, WHO, Group WB. Levels and trends in child malnutrition: Key finding of the 2023 edition. Asia-Pacific Popul J. 2023;24(2):51–78.
2. Fanzo J. The Nutrition Challenge in Sub-Saharan Africa. 2012;(January).

3. Ethiopian Public Health Institute Addis Ababa. Ethiopia Mini Demographic and Health Survey. FEDERAL DEMOCRATIC REPUBLIC OF ETHIOPIA Ethiopia. 2019.

4. UNICEF. Sub-Saharan Africa Growing up in Crisis in a World of Opportunities. Unicef [Internet]. 2021;(April). Available from: https://www.unicef.org/media/96161/file/Sub-Saharan Africa – Growing up in crisis in a world of opportunities .pdf.

5. Rajkumar AS, Gaukler C, Tilahun J. Combating Malnutrition in Ethiopia: An Evidence-Based Approach for Sustained Results [Internet]. 2011. 227 p. Available from: http://documents.worldbank.org/curated/en/456711468024616593/pdf/660480PUB0EPI10mbating0malnutrition.pdf

6. Course T, The ON, Of M, Malnutrition A. TRAINING COURSE ON THE COMPREHENSIVE MANAGEMENT OF ACUTE MALNUTRITION PROGRAM Federal Ministry of Health Addis Ababa. 2019;

7. July Y. Borena zone Health Office Out line. 2022;

8. Ethiopia MOH. Training Course on the Management of Acute Malnutrition. 2019;

9. Mamo WN, Derso T, Gelaye KA, Akalu TY. Time to recovery and determinants of severe acute malnutrition among 6 – 59 months children treated at outpatient therapeutic programme in North Gondar zone , Northwest Ethiopia : a prospective follow up study. 2019;1–8.

10. Bekalu A, Sahlu D, Tadesse AW, Asmare B, Hune Y, Tilahun B. Time to Recovery and Determinants of Uncomplicated Severe Acute Malnutrition Among 6 to 59 Months Children From the Outpatient Therapeutic Feeding Program in North Shewa Zone of Oromia Region, Ethiopia: A Prospective Follow-up Study. Nutr Metab Insights. 2022;15:117863882211069.

11. Federal Ministry of Health. National Guideline for the Management of Acute Malnutrition in Ethiopia. Natl Guidel Manag Acute Malnutrition Ethiop. 2019;(May):136.

12. FAOSTAT. Food security indicators. Food Agric Organ united Nations. 2018;(February).

13. Liben ML, Wuneh AG, Shamie R, G/her K. Factors associated with child survival in children admitted to outpatient therapeutic program at public health institutions in Afar Regional State, Ethiopia: a prospective cohort study. J Heal Popul Nutr. 2019;38(1):1–8.

14. Fikrie A, Alemayehu A, Gebremedhin S. Treatment outcomes and factors affecting time-to-recovery from severe acute malnutrition in 6-59 months old children admitted to a stabilization center in Southern Ethiopia: A retrospective cohort study. Ital J Pediatr. 2019;45(1):1–9.

15. Asres DT, Prasad RPCJ, Ayele TA. Recovery inpt IN SAM LET&Pro. 2018;1–8.

16. Trehan I, Goldbach HS, LaGrone LN, Meuli GJ, Wang RJ, Maleta KM, et al. Antibiotics as part of the management of severe acute malnutrition. Malawi Med J. 2016;28(3):123–30.

17. Teshome G, Bosha T, Gebremedhin S. Time-to-recovery from severe acute malnutrition in children 6-59 months of age enrolled in the outpatient treatment program in Shebedino, Southern Ethiopia: A prospective cohort study. BMC Pediatr. 2019;19(1):1–10.

18. Gebremedhin K, Ayele G, Boti N, Andarge E, Fikadu T. Predictors of time-To-recovery from severe acute malnutrition treated in an outpatient treatment program in health posts of Arba Minch Zuria Woreda, Gamo Zone, Southern Ethiopia: A retrospective cohort study. PLoS One. 2020;15(6 June):1–14.

19. Atnafe B, Teji K, Id R, Dingeta T. Time of recovery and associated factors of children with severe acute malnutrition treated at outpatient therapeutic feeding program in Dire Dawa , Eastern Ethiopia. 2019;569:1–16.

Supporting information

S1. Supplementary File. English version Questionnaires.

Other Element

Fig. 1.Sketch Map of Borena Zone.

Fig.2.Schematic diagram of sampling technique and procedure.

Fig.3.Types of SAM diagnosed during admission.

Fig.4 Survival probability on OTP treatment.

Fig.5.Survival status among SAM children.

Fig. 6. Survival status among SAM children received Amoxicillin.

Scanned picture.DU-IRB Ethical approval Letter

Click here to access/download;Figure;Fig. 1.Sketch Map of Borena Zone.docx

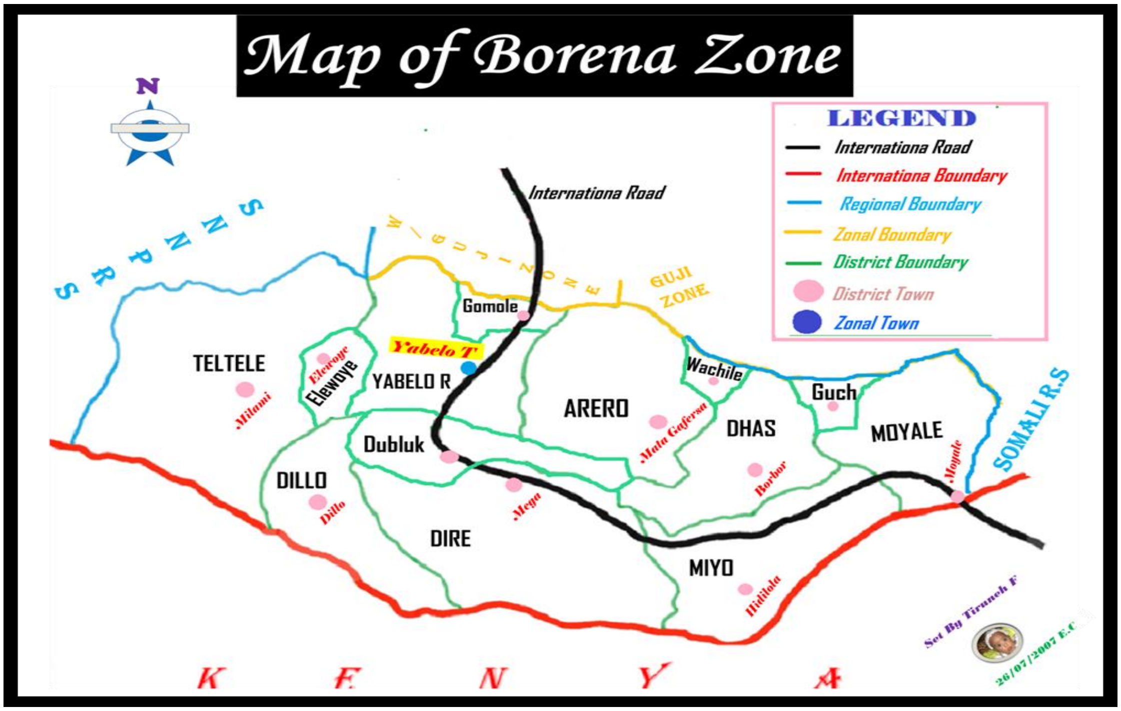

*Fig.**1**: Sketch Map of Borena Zone, Oromia Region, Southern Ethiopia, 2023 G.C*

(Source: Borena Zone Administration office)

Figure

Click here to access/download;Figure;Fig.2.Schematic diagram of sampling technique and procedure.docx

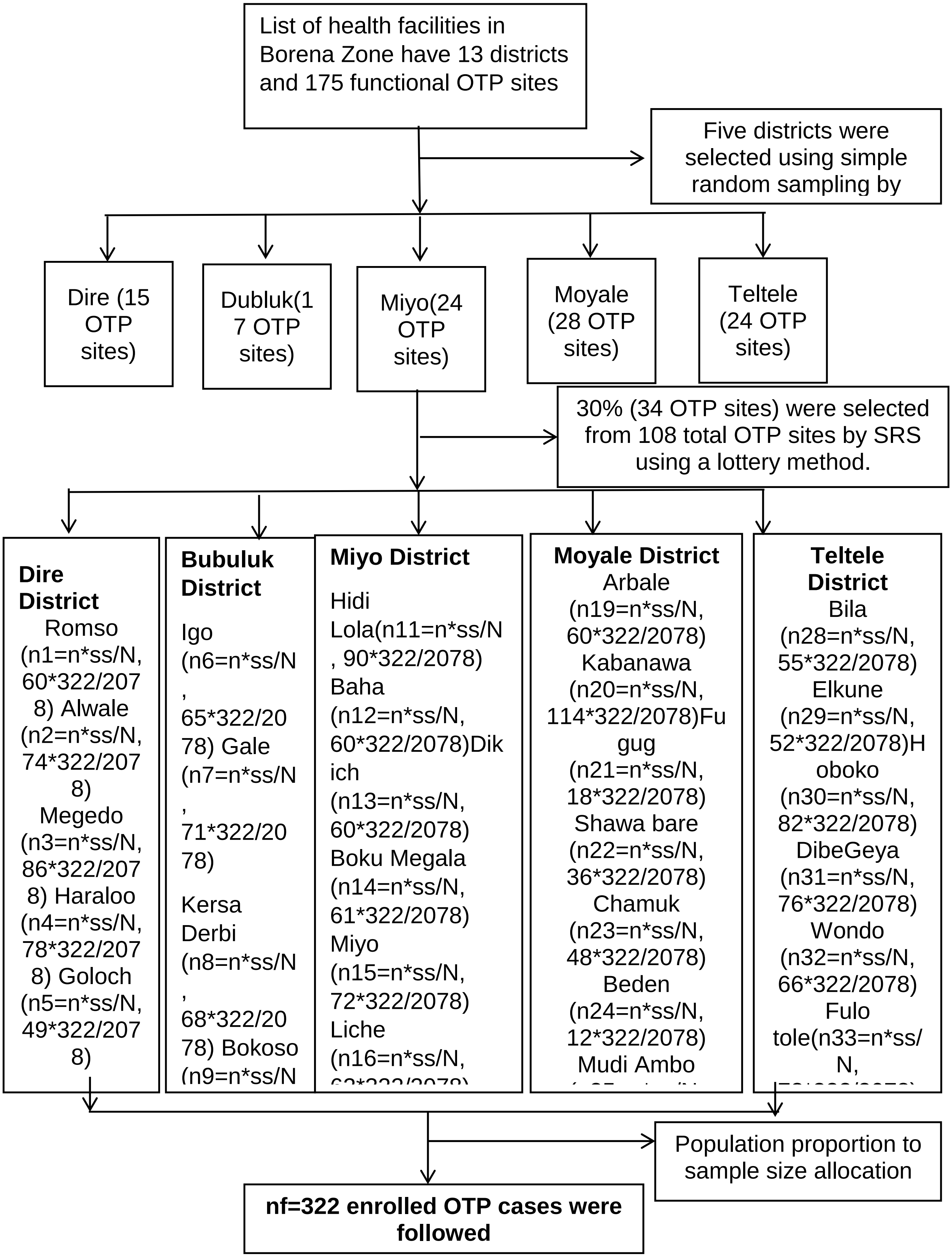

Fig.2: Schematic diagram of sampling technique and procedure of duration of recovery and predictors, among 6 to 59 months age children registered at the OTP program in Borena zone, Oromia region, Ethiopia 2023.

Click here to access/download;Figure;Fig.3.Types of SAM diagnosed during admission.docx

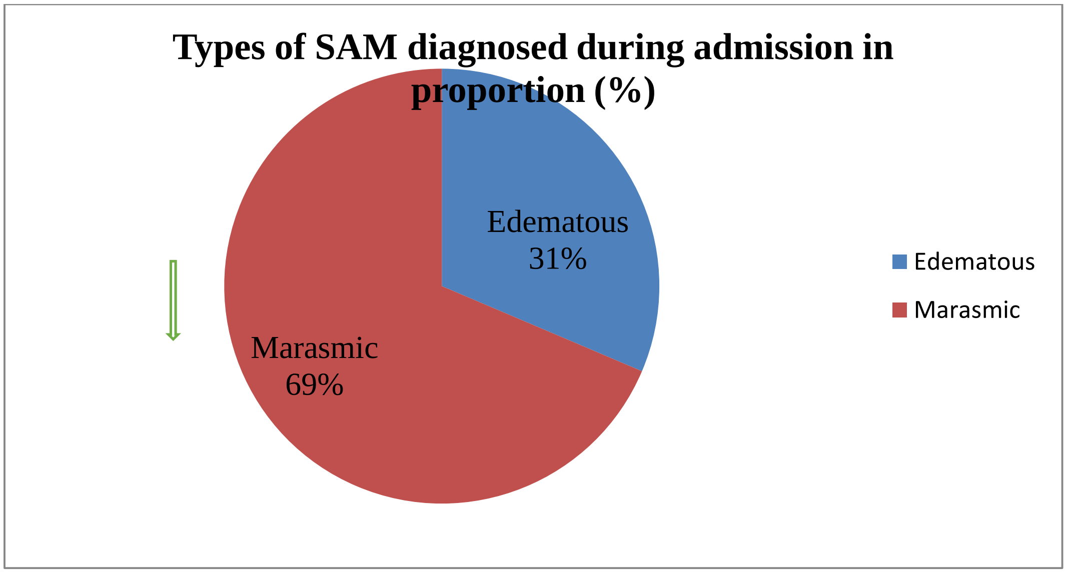

Fig.3: Types of SAM diagnosed during admission among children aged 6-59 months at OTP in Borena zone, Southern Ethiopia 2023.

Figure
Click here to access/download;Figure;Fig.4 Survival probability on OTP treatment.docx

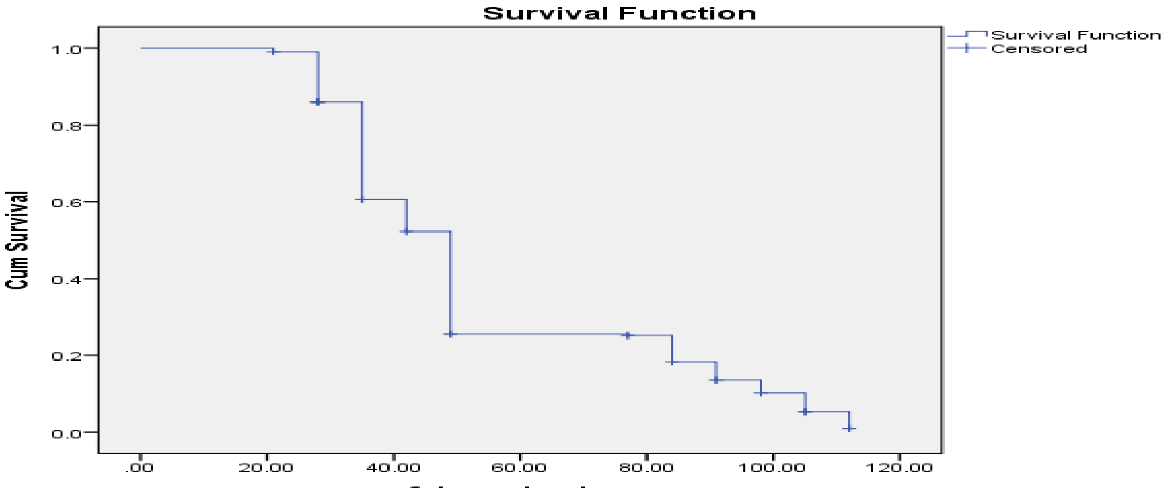

Follow up in days

Fig.4.Survival probability on OTP treatment among children 6 to 59 months of age diagnosed with SAM managed at OTP in Borena zone, Oromia, Southern Ethiopia 2023.

Figure Click here to access/download;Figure;Fig.5.Survival status among SAM children.docx

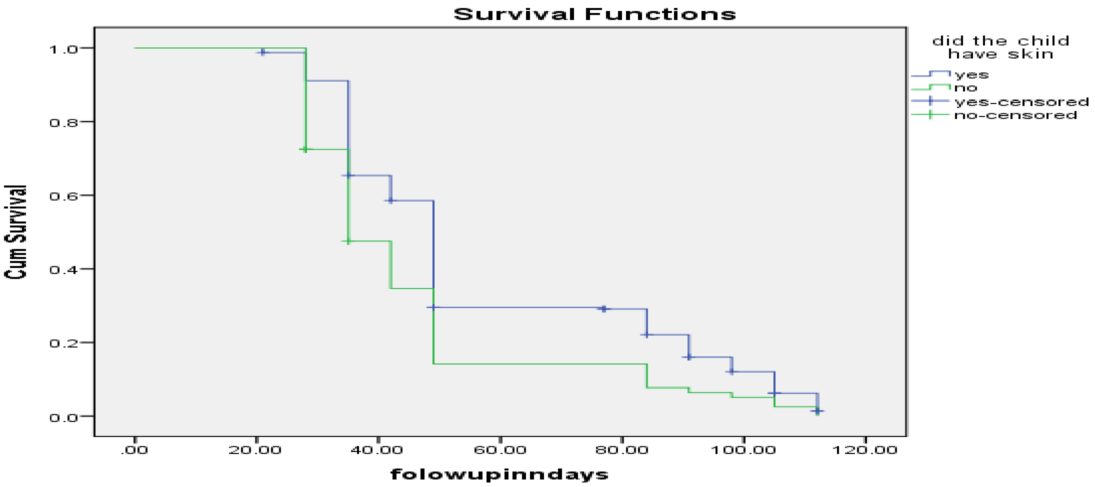

Fig.5: Survival status among SAM children 6 to 59 months of age with skin infection with managed at OTP in Borena zone, Oromia, Southern Ethiopia 2023.

Click here to access/download;Figure;Fig. 6.Survival status SAMC eceived Amoxicillin.docx

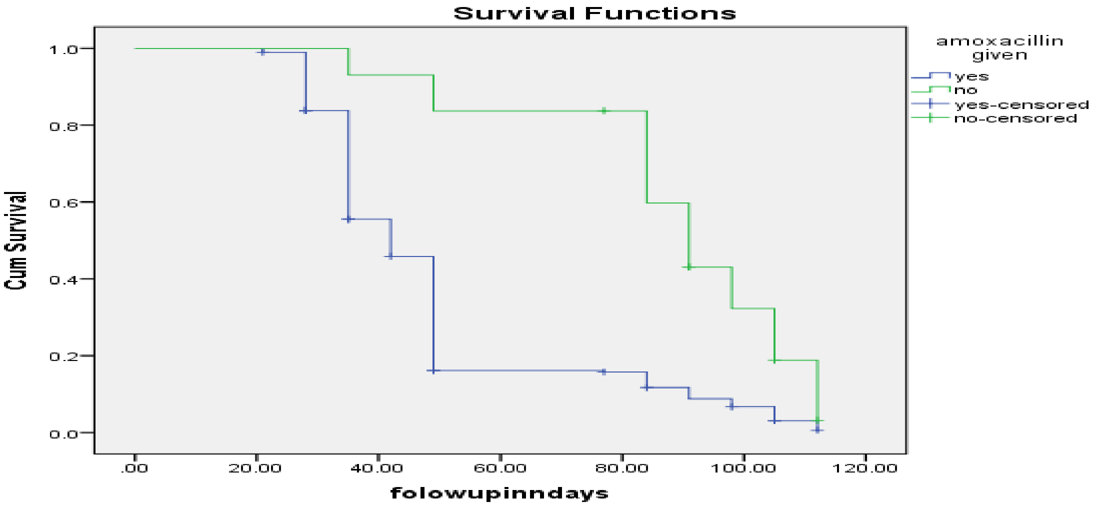

Fig. 6: Survival status among SAM children who received Amoxicillin while OTP management in Borena zone, Oromia, Southern Ethiopia 2023

Click here to access/download
Supporting Information
S1.Supplimantary file.English version questionnaire.docx
