# Supplementary material for: Recovery time and predictors of severe acute malnutrition in children aged 6–59 months via an outpatient therapeutic program in Borena zone: A prospective cohort study
Source: PLoS One. 2025 Jan 3;20(1):e0313186. doi: 10.1371/journal.pone.0313186 (PMC11698384; doi:10.1371/journal.pone.0313186)
Supplement: Attachment — Submitted filename: Response to the reviewrs.docx [file pone.0313186.s003.docx]

**Point by point response to the reviewers**

I. **Section 3: Review Comments to the Author**

**Reviewer#1: Comment to the authors and Responses given by the authors**

Please use the space provided to explain your answers to the questions above. You may also include additional comments for the author, including concerns about dual publication, research ethics, or publication ethics. (Please upload your review as an attachment if it exceeds 20,000 characters).

**Reviewer #1:**

1. Provide more details regarding the specific ethical considerations addressed in the study, such as informed consent procedures and data confidentiality measures.

**Response to Reviewer #1**

Dear Reviewer#1, We would like to express our sincere gratitude for your thorough review and valuable feedback regarding our manuscript. We appreciate the opportunity to clarify and address the concerns you raised, particularly concerning the ethical considerations associated with our study.

We would like to reiterate that prior to initiating the study, we obtained ethical approval from the Institutional Review Board (IRB) at Dila University’s College of Health Sciences and Medicine. An official letter was then sent to the Borena zone health office, which further communicated to the relevant district health offices and outpatient therapeutic program (OTP) sites. Verbal consent was obtained from the mothers or caregivers before participation, ensuring that they fully understood the purpose of the study, the procedures involved, and their right to withdraw at any time. We emphasized the confidential nature of their responses and assured them that their participation would not impact their access to health services.

To safeguard participant confidentiality, the data extraction forms did not include any identifying information of the children. Data was stored securely with limited access, employing password protection and strict data handling protocols. We used unique identification numbers linked to OTP registration to maintain data integrity while ensuring privacy. We took special care to inform caregivers clearly about the study's aims, which helped reduce any potential social desirability bias. This transparency was crucial in ensuring that caregivers provided accurate and honest information.

We have revised the manuscript to include these additional details, specifically in the methodology and ethical considerations sections (refer to the last part of the methods and material, in the Ethical Consideration sub-section on pages 12 for the updates). We believe these modifications address your concerns and strengthen the manuscript.

Thank you once again for your constructive feedback. We look forward to your further comments. Sincerely, Dr Girma Tenkolu, corresponding author.

2. It would be beneficial to provide a succinct summary of the key findings before delving into the detailed interpretation. This will help readers grasp the main findings and their significance right at the beginning of the discussion.

**Response to Reviewer #1**

Thank you, Reviewer #1, for your valuable feedback on our PLOS ONE manuscript. We appreciate your suggestion to include a succinct summary of the key findings at the beginning of the discussion, and we have revised this section accordingly.

To highlight the significance of our research immediately, we now open the discussion with a summary that includes: The study aimed to assess the median recovery time from severe acute malnutrition in 322 children in the Borena zone, Oromia region, Southern Ethiopia. Results indicated a recovery rate of 93.2%, demonstrating the effectiveness of the treatment protocols employed. The median recovery time was established at 42 days. Notably, the presence of symptoms such as diarrhea, vomiting, and edema at admission was identified as significant predictors that prolonged recovery time. Conversely, timely administration of routine medications, particularly Amoxicillin, was associated with faster recovery.

This adjustment aims to provide readers with a clear understanding of our key findings and their implications right from the outset. We believe this change will enhance the manuscript's clarity and impact.

Thank you once again for your constructive suggestions. We trust that these improvements address your concerns and strengthen our submission. For confirmation, please see the first paragraph of the discussion part of the revised manuscript starting from line No 1 up to the end. Sincerely, Dr Girma Tenkolu

**Reviewer#2: Comment to the authors and Responses given by the authors**

**Reviewer #2:**

1. Reviewing the paper titled “Recovery Time and Predictors of Severe Acute Malnutrition in Children Aged 6–59 Months via an Outpatient Therapeutic Program in Borena Zone: A Prospective Study”. The study contributes to the body of literature on the recovery time and predictors of SAM among children with unsevere conditions admitted to healthcare services.

**Response Given**

Dear Reviewer # 2

Thank you for your thoughtful and constructive feedback regarding our manuscript. We greatly appreciate the time and effort you have dedicated to reviewing our work, and we are committed to addressing your concerns thoroughly to enhance the quality of our manuscript.

We understand your observation regarding the study's contribution to the existing literature on severe acute malnutrition (SAM) and recovery time predictors. To clarify and amplify the relevance of our research, we will include a more comprehensive introduction that highlights the current state of knowledge on SAM recovery and emphasizes the importance of understanding recovery times and influencing factors, particularly in populations with varying admissions criteria. This will better contextualize our findings within the broader field of malnutrition research.

We will ensure that our results are communicated clearly, specifically detailing how the predictors identified (diarrhea, vomiting, edema, and timely medication) relate to recovery times. We believe that a more in-depth discussion of our findings will better illustrate the implications of our study for treatment strategies and public health policies in similar settings.

In response to your suggestion, we will include a section discussing potential directions for future research, such as exploring additional factors influencing recovery times or conducting similar studies in different regions or populations. This addition will provide valuable insights for researchers and practitioners aiming to address SAM more effectively.

We have revised the manuscript to incorporate these suggestions and improve clarity, coherence, and overall quality. We hope that these changes address your concerns and further establish the significance of our findings within the field.

Thank you once again for your valuable feedback. We look forward to your further comments and hope to fulfill the publication requirements of PLOS ONE.

Sincerely, Dr Girma Tenkolu , Corresponding author

2. Abstract: well written and provide a clear overview of the study. The conclusion should clearly mention that the results are only generalizable to children with SAM and to those who access healthcare services rather than every child with SAM. Those who seek health services have more severe symptoms.

**Response Given:** Dear Reviewer # 2

Thank you for your constructive feedback on our manuscript. We appreciate the time you took to review our work and your insightful comments.

We acknowledge your point regarding the generalizability of our findings. As per your suggestion, we have revised the conclusion to clarify that the results are specific to children experiencing severe acute malnutrition (SAM) who access healthcare services. In the revised manuscript, we emphasize that the outcomes might not be applicable to all children with SAM, particularly those who do not seek treatment or have less severe symptoms.

In the updated conclusion, we explicitly state: “These results are generalizable only to children with severe acute malnutrition who access healthcare services and may not reflect the broader population of all children with SAM, particularly those presenting with milder symptoms or those not seeking treatment.” This statement aims to ensure that readers appreciate the context and limitations of our findings.

We believe that this revision enhances the clarity and accuracy of our conclusions. Thank you once again for your invaluable feedback. We trust that these modifications align with your expectations and contribute to the overall quality of our manuscript. We look forward to your further comments.

Sincerely, Dr Girma Tenkolu , Corresponding author

3. Introduction: The study provides an overview of the literature, focusing on Ethiopia. Since Marasmus and edematous was mentioned later in the results, it would be good to define them and explain the difference.

Here’s a revised version that includes definitions and explanations of marasmus and edematous malnutrition:

**Response Given:** Dear Reviewer # 2

Thank you for your thoughtful feedback on our manuscript. We appreciate the time and effort you have dedicated to reviewing our work.

We acknowledge your suggestion regarding the inclusion of definitions and explanations for marasmus and edematous malnutrition in the Introduction section. To enhance the clarity of our manuscript and ensure that readers have a comprehensive understanding of these concepts, we have included the following definitions and distinctions: In the context of severe acute malnutrition (SAM), two primary forms are recognized: marasmus and kwashiorkor (edematous malnutrition). Marasmus is characterized by severe weight loss and muscle wasting due to inadequate energy intake, resulting in a noticeable loss of fat and muscle tissue. In contrast, kwashiorkor is primarily associated with protein deficiency and is often recognizable by the presence of edema, which causes swelling in the body, particularly in the abdomen and lower extremities. Understanding these distinctions is crucial for appropriate diagnosis and management of malnutrition in affected populations.

We believe that this addition enriches the manuscript by providing readers with essential background information that will enhance their understanding of the results presented later in the study.

Thank you again for your valuable insights. We trust that these changes address your concerns and improve the overall quality of our manuscript. We look forward to your further comments.

Sincerely, Sincerely, Dr Girma Tenkolu , Corresponding author

4. **Methods:**
i. Mention clearly what OTP intervention entails at the very beginning of the paper (types, doses etc)
   Precise whether the study sample have received a complementary treatment (other than OTP or from other services) to treat SAM. Mention if they were included in the sample or accounted for in the analysis.

**Response given : D**ear Reviewer#2

We appreciate your valuable feedback and the opportunity to clarify the treatment regimen for the study sample. To address your inquiry, we confirm that all children included in our study exclusively received Ready-to-Use Therapeutic Food (RUTF) as part of the Outpatient Therapeutic Feeding Program (OTP). No complementary treatments were provided to any participants during the study period, nor were any children receiving additional therapies included in our analysis. This focus allowed us to assess the effectiveness of the OTP in treating Severe Acute Malnutrition (SAM) without the confounding effects of other interventions. We hope this response sufficiently addresses your concerns and clarifies the methodology of our study. Thank you for your constructive suggestions, which have helped enhance the clarity and rigor of our manuscript. Sincerely, Dr Girma Tenkolu

i. The 117 inclusion criteria here is unclear: “Specifically, children who met the 117 inclusion criteria and were enrolled in a randomly chosen OTP service site during the 118 study period were considered as study participants”

**Response given : D**ear Reviewer#2

Thank you for your valuable feedback regarding our manuscript submitted to PLOS ONE. We appreciate the reviewers’ insights, which have helped us enhance the clarity and rigor of our study.

In response to the concern about the clarity of our inclusion criteria, we acknowledge that the phrasing may have caused some confusion. To clarify, children eligible for inclusion in our study were defined by the following criteria:

- Participants must be between 6 to 59 months old.
- All children must have been diagnosed with uncomplicated severe acute malnutrition as per the World Health Organization guidelines.
- Children must have passed an appetite test prior to enrollment.
- Eligible participants displayed bilateral pitting edema and a Weight-for-Length/Height (WFL/WFH) measurement of less than -3 Z-scores, indicating the severity of malnutrition without any accompanying medical complications.
- Children were enrolled from randomly selected Outpatient Treatment Program (OTP) service sites during the study period, which lasted from March 1 to March 30, 2023.

We have amended the manuscript to include a more detailed description of the inclusion criteria, making it explicit for readers. This addition should enhance the transparency of our methodology and allow for a better understanding of the study population.

Thank you again for your constructive feedback. We are committed to addressing all reviewer concerns and improving our manuscript for publication. For more confirmation, please refer the method section on page 7, in the sub-section of Study population and Inclusion and Exclusion Criteria, line No 1 to end. Sincerely, Dr Girma Tenkolu

ii. Is there a difference in the dose administered to children?

Response given

Dear Reviewer#2, Thank you for your insightful feedback regarding our manuscript submitted to PLOS ONE. We appreciate your attention to detail and your valuable suggestions for improving our work.

In response to the question regarding differences in the doses administered to children, we would like to clarify the following:

- The treatment protocols followed in our study adhered to the standardized guidelines established by the World Health Organization for the management of severe acute malnutrition in children. The dosage of Amoxicillin, specifically, was determined based on the weight of the children and followed the recommended guidelines for treatment of infections common in this population.
- The doses were carefully calculated for each child based on their weight and clinical condition at the time of treatment. For instance, children typically received 20-40 mg/kg/day of Amoxicillin divided into two doses. This approach ensures that each child receives an appropriate dosage tailored to their specific needs.
- We maintained detailed records of the medication administered, including dosages, to ensure compliance with treatment protocols and the ability to assess any impact on recovery times.

To enhance clarity in our manuscript, we will include additional details regarding the dosing protocols for Amoxicillin and other medications used in our study. We believe this will address your concerns and provide a clearer understanding of our methodology. Thank you once again for your constructive comments. We are dedicated to refining our manuscript in response to all reviewer feedback and ensuring it meets the publication standards. For more confirmation, please refer the method section on page 12-13, in the sub-section of Independent variables:, line No 15 to end. Sincerely, Dr Girma Tenkolu

iii. How was the HHFD calculated? is it the sum of the consumption of each of the food groups? Precise how this has been calculated and if any weights have been used for highly nutritious food groups. For HHFD, how did you decide on the cutoff points for household food diversity score? Please add references or mention if the median or mean has been used

**Response given : D**ear Reviewer#2

Thank you for your insightful questions regarding the calculation of the Household Food Diversity Score (HHFD) within our study. We appreciate your attention to detail, as it aids in enhancing the clarity of our methodology.

The HHFD was assessed using a standardized tool that evaluates the consumption of seven distinct food groups. Each household was asked about their dietary intake over a specified period, and the scores were subsequently computed using SPSS software. Households were categorized based on their scores, with those achieving a score of 4.5 or higher classified as having medium dietary diversity, while those scoring below 4.5 were considered to have poor dietary diversity. To clarify, the score reflects the cumulative consumption across the specified food groups without applying specific weights to highly nutritious food categories. This approach ensures a comprehensive assessment of dietary diversity at the household level.

We hope this explanation clarifies the methodology used in calculating the HHFD, and we will incorporate these details into the manuscript to enhance its overall rigor and transparency.

Thank you for your valuable feedback. We are committed to addressing all reviewer concerns to ensure our manuscript meets the highest standards for publication. Sincerely, Dr Girma Tenkolu,

iv. Add a footnote on what does ‘other vegetables’ entail

**Response given : D**ear Reviewer#2

Thank you for your valuable feedback regarding the terminology used in our manuscript. We appreciate your attention to detail and the opportunity to clarify our definitions.

In response to your request, we will add a footnote to specify that “other vegetables” refers to a variety of non-starchy vegetables, including but not limited to leafy greens, tomatoes, carrots, cauliflower, and other similar produce. This category encompasses vegetables that are not classified under common types, such as root vegetables or legumes, to provide a more comprehensive understanding of the dietary diversity assessed in our study. (refer to result section, in the Household Food Diversity status of Caretakers/mothers and children sub-section, at the end of the table, on pages 19 for the updates).

We believe this additional detail will enhance the clarity of our findings and contribute to the overall rigor of the manuscript. Thank you again for your constructive comments. We are committed to addressing all reviewer concerns to ensure our work is clear and impactful. Sincerely, Dr Girma Tenkolu.

5. Ethical consideration: provide further details on how children were matched at baseline and follow-up visits since the data was sent for analysis anonymously

**Response Given :Dear Reviewer#2,**

Thank you for your insightful inquiry regarding the ethical considerations in our study, specifically concerning how children were matched at baseline and follow-up visits despite the anonymous data analysis.

To clarify, we utilized a unique identification number assigned to each child during the Outpatient Therapeutic Program (OTP) registration. This identification number was essential for linking baseline information with follow-up outcomes effectively. Additionally, we ensured that the study’s objectives were clearly communicated to the mothers or caregivers, which was pivotal in managing social desirability bias during data collection. (refer to Methods and material section, in the ethical clearance sub-section, at the end of the method partfor the updates).

We appreciate your attention to this important aspect of our methodology and will incorporate this clarification into the manuscript. Thank you for your valuable feedback, and we are committed to addressing all reviewer concerns to enhance the quality of our work. Sincerely, Dr Girma Tenkolu

6. Discussion: elaborate on how you addressed social desirability bias in the study, it is unclear.

Response Given: Dear Reviewer'#2,

Thank you for your thoughtful feedback regarding our discussion of social desirability bias in the study. We appreciate your attention to this important aspect of our research methodology.

To mitigate social desirability bias, we employed several strategic approaches during the data collection phase: The researchers carefully explained the goals of the study to mothers and caregivers, emphasizing that the primary aim was to assess treatment efficacy and improve health outcomes for children with severe acute malnutrition. This transparency was intended to foster a collaborative environment where participants felt comfortable providing honest responses. Participants were assured that their responses would be kept confidential and would only be used for research purposes. By reinforcing that individual answers would not affect the care their children received, we aimed to alleviate any concerns about judgment or repercussions. Data collectors received comprehensive training on how to interview participants in a manner that was non-judgmental and respectful. They were encouraged to establish rapport with caregivers, which facilitated open dialogue and encouraged honest responses. Where feasible, we included questions framed in a way that minimized the pressure on participants to give socially desirable answers. This approach aimed to gather more accurate reflections of their experiences and perceptions. In some instances, we engaged in follow-up discussions with caregivers to confirm and clarify certain responses, ensuring that the data collected accurately reflected their views and circumstances.

We recognize the importance of acknowledging and addressing biases in our research, and we will expand upon these points in the revised discussion section of the manuscript. Thank you once again for your constructive feedback. We are committed to enhancing the manuscript and ensuring that all aspects of our methodology are clearly articulated. Sincerely, Dr Girma Tenkolu, Corresponding author

7. Conclusion: This study must clearly state that the results are generalizable to children with uncomplicated severe acute malnutrition who accessed health facilities.

**Response given : D**ear Reviewer#2

Thank you for your valuable feedback. To address your concern regarding the generalizability of the study results, we acknowledge the necessity of clearly stating that the findings are applicable to children with uncomplicated severe acute malnutrition who have accessed health facilities. In response to this point, we have revised the conclusion section of the manuscript to explicitly state that the study outcomes are specifically relevant to this target population. By emphasizing this limitation, we aim to ensure clarity and precision in conveying the applicability of our findings. We appreciate your guidance in enhancing the transparency and accuracy of our research, and we trust that this clarification adequately addresses the concern raised.

Thank you for your consideration. Warm regardsDr Girma Tenkolu

8. NB: Define the abbreviation when it appears for the first time in the text (e.g. HEW, SECA, etc).

Response to Reviewer Comments,

We would like to thank the reviewers for their valuable feedback and for allowing us the opportunity to improve our manuscript.

To address the request regarding the definition of abbreviations, we have made the following changes to our manuscript:

- We have clarified that "HEW" stands for **Health Extension Workers**, who play a vital role in the healthcare system in our study context.
- We have defined "SECA" as follows: **SECA (Société Européenne de Cardiologie et d'Athérosclérose)** is a standardized tool for measuring anthropometric data, such as weight and height, that was utilized in our study to assess the nutritional status of children over two years. We also noted that this tool undergoes regular calibration and accuracy checks to ensure precision in measurements.

These definitions have been appropriately placed at their first mention in the manuscript to enhance clarity.

Thank you again for your constructive comments, and we hope the revisions meet your expectations.

Sincerely, Dr Girma Tenkolu, corresponding author
