# Supplementary material for: Recovery time and predictors of severe acute malnutrition in children aged 6–59 months via an outpatient therapeutic program in Borena zone: A prospective cohort study
Source: PLoS One. 2025 Jan 3;20(1):e0313186. doi: 10.1371/journal.pone.0313186 (PMC11698384; doi:10.1371/journal.pone.0313186)
Supplement: Attachment — Submitted filename: The 3rd Point by point respons letter to PloseoneF.docx [file pone.0313186.s004.docx]

**Point by point letter**

**Manuscript Submission ID**: PONE-D-24-07649R1

**Title of the manuscript:** Recovery Time and Predictors of Severe Acute Malnutrition in Children Aged 6–59 Months via an Outpatient Therapeutic Program in Borena Zone: *A Prospective Cohort Study.*
Dear Dr. Bune,
Thank you for submitting your manuscript to PLOS ONE. After careful consideration, we feel that it has merit but does not fully meet PLOS ONE’s publication criteria as it currently stands. Therefore, we invite you to submit a revised version of the manuscript that addresses the points raised during the review process.
Please submit your revised manuscript by Nov 02 2024 11:59PM. If you will need more time than this to complete your revisions, please reply to this message or contact the journal office at [plosone@plos.org](mailto:plosone@plos.org). When you're ready to submit your revision, log on to <https://www.editorialmanager.com/pone/> and select the 'Submissions Needing Revision' folder to locate your manuscript file.

Please include the following items when submitting your revised manuscript:

- A rebuttal letter that responds to each point raised by the academic editor and reviewer(s). You should upload this letter as a separate file labeled 'Response to Reviewers'.
- A marked-up copy of your manuscript that highlights changes made to the original version. You should upload this as a separate file labeled 'Revised Manuscript with Track Changes'.
- An unmarked version of your revised paper without tracked changes. You should upload this as a separate file labeled 'Manuscript'.

If you would like to make changes to your financial disclosure, please include your updated statement in your cover letter. Guidelines for resubmitting your figure files are available below the reviewer comments at the end of this letter.

If applicable, we recommend that you deposit your laboratory protocols in [protocols.io](http://protocols.io/) to enhance the reproducibility of your results. Protocols.io assigns your protocol its own identifier (DOI) so that it can be cited independently in the future. For instructions see: [https://journals.plos.org/plosone/s/submission-guidelines#loc-laboratory-protocols](https://c05y1x9s.r.us-east-2.awstrack.me/L0/https:%2F%2Fjournals.plos.org%2Fplosone%2Fs%2Fsubmission-guidelines%23loc-laboratory-protocols/1/010f01920390416d-aa834fa3-4bff-49d2-b0d2-24996b1a0fad-000000/KR0xIaI0lEcjsIi7CY6xR9rwMW4=177). Additionally, PLOS ONE offers an option for publishing peer-reviewed Lab Protocol articles, which describe protocols hosted on [protocols.io](http://protocols.io/). Read more information on sharing protocols at [https://plos.org/protocols?utm_medium=editorial-email&utm_source=authorletters&utm_campaign=protocols](https://c05y1x9s.r.us-east-2.awstrack.me/L0/https:%2F%2Fplos.org%2Fprotocols%3Futm_medium=editorial-email%26utm_source=authorletters%26utm_campaign=protocols/1/010f01920390416d-aa834fa3-4bff-49d2-b0d2-24996b1a0fad-000000/U1JT7mG7b3wBI0j-4wvTICYk6ek=177).

We look forward to receiving your revised manuscript.

Kind regards,

Ammal Mokhtar Metwally, Ph.D (MD)
Academic Editor
PLOS ONE

**Journal Requirements:**
Please review your reference list to ensure that it is complete and correct. If you have cited papers that have been retracted, please include the rationale for doing so in the manuscript text, or remove these references and replace them with relevant current references. Any changes to the reference list should be mentioned in the rebuttal letter that accompanies your revised manuscript. If you need to cite a retracted article, indicate the article’s retracted status in the References list and also include a citation and full reference for the retraction notice.
**Additional Editor Comments:** The manuscript is interested meanwhile, the reviewers have raised a number of points which we believe would improvethe manuscript and may allow a revised version to be published in PLOS one.

**Response to Academic Editor of PLOS ONE**

**Dear Ammal Mokhtar Metwally,**

**Ph.D (MD), Academic Editor, PLOS ONE**

Thank you for your feedback and the opportunity to revise our manuscript. We appreciate the constructive comments and the invitation to submit a revised version of our work. Below, we address each of the journal’s requirements and comments point by point.

**Regarding the issues of Journal Requirements:**

1. **Complete and Correct Reference List:**

We have thoroughly reviewed the reference list and corrected any inaccuracies. Any papers that have been retracted have been indicated as such in the text of the revised manuscript. Additionally, we have provided a rationale for including these references, where applicable, and replaced outdated references with more current and relevant studies.

2. **Regarding the issues of Additional Editor Comments:**

**Points Raised by Reviewers:** The manuscript has been revised in response to reviewer comments, particularly regarding the Household Food Security (HHFS) score and its cutoff point of 4.5. The Household Food Diversity Score (HHFD) measures dietary diversity by assessing the variety of food groups consumed, with a focus on nutritional quality. In the study, HHFD was evaluated using a standardized tool analyzing seven distinct food groups, categorizing households into medium or poor dietary diversity based on the 4.5 score threshold. This cutoff aligns with existing literature that effectively distinguishes between food secure and food insecure households and reflects the median score observed in the study population. Adopting this cutoff enhances the relevance of the findings to food security assessments, particularly for vulnerable populations, and supports interventions addressing food insecurity and severe acute malnutrition.

3. **Regarding the issues of Additional Items for Submission:**
   a. **Rebuttal Letter:** As per your instructions, this letter serves as our rebuttal, responding to the editor's and reviewers' concerns.
   b. **Marked-up Copy of the Manuscript:** We have uploaded a marked-up copy of the manuscript highlighting all changes made, labeled as 'Revised Manuscript with Track Changes'.
   c. **Unmarked Version of the Manuscript:** An unmarked version of the manuscript, labeled 'Manuscript', has also been submitted.
4. **Financial Disclosure:** No changes to the financial disclosure are necessary, as there are no updates regarding funding or conflicts of interest.

We believe that these revisions substantially improve the manuscript and address all concerns raised. Thank you for your consideration, and we look forward to your feedback on our revised submission.

**Kind regards,**

**Dr.Girma Tenkolu Bune, the cooresponding Author of the manuscript.**

**Dilla University**

**Email. girmatbune@gmail.com**

**Reviewers' comments:**

**Reviewer's Responses to Questions**

**Comments to the Author**

1. If the authors have adequately addressed your comments raised in a previous round of review and you feel that this manuscript is now acceptable for publication, you may indicate that here to bypass the “Comments to the Author” section, enter your conflict of interest statement in the “Confidential to Editor” section, and submit your "Accept" recommendation.

Reviewer #2: All comments have been addressed

2. Is the manuscript technically sound, and do the data support the conclusions?

The manuscript must describe a technically sound piece of scientific research with data that supports the conclusions. Experiments must have been conducted rigorously, with appropriate controls, replication, and sample sizes. The conclusions must be drawn appropriately based on the data presented.

Reviewer #2: Yes

3. Has the statistical analysis been performed appropriately and rigorously?

Reviewer #2: Yes

4. Have the authors made all data underlying the findings in their manuscript fully available?

The [PLOS Data policy](http://c05y1x9s.r.us-east-2.awstrack.me/L0/http:%2F%2Fwww.plosone.org%2Fstatic%2Fpolicies.action%23sharing/1/010f01920390416d-aa834fa3-4bff-49d2-b0d2-24996b1a0fad-000000/VgdLgw5TiYhJO8qLDfBlau48XHY=177) requires authors to make all data underlying the findings described in their manuscript fully available without restriction, with rare exception (please refer to the Data Availability Statement in the manuscript PDF file). The data should be provided as part of the manuscript or its supporting information, or deposited to a public repository. For example, in addition to summary statistics, the data points behind means, medians and variance measures should be available. If there are restrictions on publicly sharing data—e.g. participant privacy or use of data from a third party—those must be specified.

Reviewer #2: Yes

5. Is the manuscript presented in an intelligible fashion and written in standard English?

PLOS ONE does not copyedit accepted manuscripts, so the language in submitted articles must be clear, correct, and unambiguous. Any typographical or grammatical errors should be corrected at revision, so please note any specific errors here.

Reviewer #2: Yes

6. Review Comments to the Author

Please use the space provided to explain your answers to the questions above. You may also include additional comments for the author, including concerns about dual publication, research ethics, or publication ethics. (Please upload your review as an attachment if it exceeds 20,000 characters)

Reviewer #2: Thank you to the authors for taking the time to respond to the comments point-by-point. The manuscript now looks great and addresses all the concerns.

**I have one minor comment:** Could you please specify why 4.5 was used as a cutoff point for HHFS? Was this choice based on literature, or did it align with the mean/median in your data? if it is based on the literature, provide references.

Best of luck with the publication of your paper.

7. PLOS authors have the option to publish the peer review history of their article ([what does this mean?](https://c05y1x9s.r.us-east-2.awstrack.me/L0/https:%2F%2Fjournals.plos.org%2Fplosone%2Fs%2Feditorial-and-peer-review-process%23loc-peer-review-history/1/010f01920390416d-aa834fa3-4bff-49d2-b0d2-24996b1a0fad-000000/8x1nEdzarTxrgCmpPtSHUpr5NDM=177)). If published, this will include your full peer review and any attached files.


If you choose “no”, your identity will remain anonymous but your review may still be made public.


**Do you want your identity to be public for this peer review?** For information about this choice, including consent withdrawal, please see our [Privacy Policy](https://c05y1x9s.r.us-east-2.awstrack.me/L0/https:%2F%2Fwww.plos.org%2Fprivacy-policy/1/010f01920390416d-aa834fa3-4bff-49d2-b0d2-24996b1a0fad-000000/ABy77x6s1sMCx8auveMMtZiWNPY=177).

Reviewer #2: No

[NOTE: If reviewer comments were submitted as an attachment file, they will be attached to this email and accessible via the submission site. Please log into your account, locate the manuscript record, and check for the action link "View Attachments". If this link does not appear, there are no attachment files.]

While revising your submission, please upload your figure files to the Preflight Analysis and Conversion Engine (PACE) digital diagnostic tool, [https://pacev2.apexcovantage.com/](https://c05y1x9s.r.us-east-2.awstrack.me/L0/https:%2F%2Fpacev2.apexcovantage.com%2F/1/010f01920390416d-aa834fa3-4bff-49d2-b0d2-24996b1a0fad-000000/hY5y4cIb6wLmfbX5_eqbbDa45lw=177). PACE helps ensure that figures meet PLOS requirements. To use PACE, you must first register as a user. Registration is free. Then, login and navigate to the UPLOAD tab, where you will find detailed instructions on how to use the tool. If you encounter any issues or have any questions when using PACE, please email PLOS at [figures@plos.org](mailto:figures@plos.org). Please note that Supporting Information files do not need this step.

Here’s a point-by-point response letter addressing Reviewer #2's comments on your manuscript. This format will help you clearly communicate how you have addressed their question and comments.

**Responses given to the Review Comments**

Responses Review Comments to the Author# 6 to the specific minor comment: Could you please specify why 4.5 was used as a cutoff point for HHFS? Was this choice based on literature, or did it align with the mean/median in your data? if it is based on the literature, provide references.

**Dear Reviewer #2,**

Thank you for your thoughtful review and for acknowledging the improvements made to our manuscript. We appreciate your feedback and have addressed your comment regarding the cutoff point for the Household Food Security (HHFS) measure.

We are grateful for your request for clarification on the use of 4.5 as a cutoff point for the HHFS. In the revised manuscript, we have provided a detailed explanation in the Methods section. This cutoff was selected based on its alignment with existing literature, which indicates that this threshold effectively distinguishes between food secure and food insecure households (relevant references are now included in the Methods and Materials section, on the sub topic of Operational definition section, Page 12-14, Paragraph 2, starting line Nu 8). Additionally, this cutoff closely corresponds to the median score observed in our data, ensuring that our analysis is pertinent to our population while maintaining consistency with established research in this field.

We appreciate your careful consideration of our work and your encouragement regarding the publication of our paper. We believe these additions enhance the clarity and rigor of our manuscript.

Thank you once again for your constructive feedback.

**Kind regards,**

**Dr. Girma Tenkolu Bune, Corresponding Author**

**Dilla University**

**Email:** [**girmatbune@gmail.com**](mailto:girmatbune@gmail.com)
